# Eczema phenotypes and risk of allergic and respiratory conditions in school age children

**DOI:** 10.1186/s13601-020-0310-7

**Published:** 2020-02-19

**Authors:** Chen Hu, Tamar Nijsten, Evelien R. van Meel, Nicole S. Erler, Christophe Piketty, Nicolette W. de Jong, Suzanne G. M. A. Pasmans, Johan C. de Jongste, Liesbeth Duijts

**Affiliations:** 1 The Generation R Study Group, Erasmus MC, University Medical Center Rotterdam, Rotterdam, The Netherlands; 2 Department of Dermatology, Erasmus MC, University Medical Center Rotterdam, Rotterdam, The Netherlands; 3 Department of Pediatrics, Erasmus MC, University Medical Center Rotterdam, Rotterdam, The Netherlands; 4 Department of Biostatistics, Erasmus MC, University Medical Center Rotterdam, Rotterdam, The Netherlands; 5 CUTIS (Clinical Unit for Tests and Imaging of Skin), Evaluation Department, Nestlé Skin Health/Galderma Research and Development, Sophia-Antipolis, France; 6 Department of Internal Medicine, Division of Allergology & Clinical Immunology, Erasmus MC, University Medical Center Rotterdam, Rotterdam, The Netherlands

**Keywords:** Eczema, Birth cohort, Child, Asthma, Allergy

## Abstract

**Background:**

Eczema phenotypes based on eczema onset and persistence might better identify groups prone to allergic and respiratory conditions than a binary definition of eczema. We examined the associations of childhood eczema phenotypes with allergic sensitization, allergy, asthma and lung function at school age.

**Methods:**

This study among 4277 children was embedded in a multi-ethnic population-based prospective cohort study. Five eczema phenotypes (never, early transient, mid-transient, late transient, persistent) based on parental-reported physician-diagnosed eczema from age 6 months until 10 years were identified. At age 10 years, allergic sensitization was measured by skin prick tests, physician-diagnosed allergy and asthma by parent-reported questionnaires, and lung function by spirometry. Adjusted linear, logistic and multinomial regression models were applied.

**Results:**

Compared with never eczema, all eczema phenotypes were associated with increased risks of asthma (odds ratios (OR) range (95% confidence interval): 2.68 (1.58, 4.57) to 11.53 (6.65, 20.01)), food and inhalant allergic sensitization (1.72 (1.25, 2.36) to 12.64 (7.20, 22.18)), and physician-diagnosed inhalant allergy (1.92 (1.34, 2.74) to 11.91 (7.52, 18.86)). Strongest effect estimates were observed of early and persistent eczema with the risk of physician-diagnosed food allergy (OR 6.95 (3.76, 12.84) and 35.05 (18.33, 70.00), respectively) and combined asthma and physician-diagnosed allergy (7.11 (4.33, 11.67) and 29.03 (15.27, 55.22), respectively). Eczema phenotypes were not associated with lung function measures.

**Conclusion:**

Eczema phenotypes were differentially associated with risks of respiratory and allergic conditions in school-aged children. Children with early transient and persistent eczema might benefit from more intense follow-up for early identification and treatment of asthma and allergies.

## Background

Childhood eczema is a chronic disease with variable onset and persistence over time. The prevalence of eczema is up to 25% in infancy and diminishes over time [1]. Eczema is strongly associated with asthma and allergic sensitization [2]. It has been suggested that children with eczema and food allergies in early life develop asthma and allergic rhinitis in later life, which has been referred to as the atopic march [3]. However, previous results of longitudinal cohorts only found a small proportion of children with eczema that follow this atopic march [4]. This might partly be explained by the definition of eczema used in these studies. In recent years, eczema phenotypes have been introduced in epidemiologic research to replace the binary definition of eczema, as they incorporate the variability in age of onset and persistence of eczema, and therefore allow identification of specific underlying risk factors which can be used to optimize personalized preventative strategies and improve public health [5]. Also, eczema phenotypes could better identify children that may be at risk for developing asthma and allergy. Results of previous studies using longitudinal birth cohorts showed that all identified eczema phenotypes in early life were associated with up to sevenfold increased risks of asthma and allergy in later life, compared to the never eczema phenotype [6, 7]. The strongest association was observed for the persistent eczema phenotype in relation to asthma and allergy. However, the eczema phenotypes are not yet determined in non-Caucasian children, related to lung function or comprehensive allergy outcomes in older childhood.

Therefore, we examined in a multi-ethnic population-based prospective cohort of 4277 children the associations of eczema phenotypes from birth until 10 years with lung function, asthma, allergic sensitization, and allergy at school-age.

## Methods

### Design

This study was embedded in the Generation R Study, a population-based prospective cohort study from early fetal life onwards in Rotterdam, the Netherlands [8]. The study has been approved by the Medical Ethical Committee of the Erasmus MC University Medical Centre in Rotterdam. Written informed consent was obtained from parents or legal guardians. Children were excluded from the current analyses if information was missing on physician-diagnosed eczema for more than 3 time points and if information on lung function, asthma and allergic sensitization were missing. A total of 4277 children were included for the current analyses (Additional file 1: Figure S1).

### Eczema phenotypes

Information on eczema was obtained from parental-reported questionnaires at the age of 6 months, and 1, 2, 3, 4 and 10 years (‘Was your child diagnosed with eczema in the last 6 months/last year by a general practitioner or physician in the hospital?’) [9]. As previously described, in children with available data on at least 3 time points between age 6 months to 10 years, latent class growth analysis was used to assign children to their latent classes based on their respective posterior probabilities [10]. Five eczema phenotypes were identified based on the various eczema trajectories: never, early transient, mid-transient, late transient and persistent eczema (Additional file 1: Figure S2). Data on ever eczema was collected by parental-reported questionnaires at 10 years of age (‘Has your child ever had eczema diagnosed by a doctor?’).

### Lung function, asthma and allergy

Children visited the research center at a median age of 9.7 years (2.5–97.5th percentile range 9.3–10.3 years). Information on lung function was measured by spirometry and included forced expiratory volume in 1 second (FEV_1_), forced vital capacity (FVC), FEV_1_/FVC, and forced expiratory flow after exhaling 75% of FVC (FEF_75_). Lung function measures were converted into sex-, height-, age-, and ethnicity-adjusted z-scores [11, 12]. Information on current asthma, and physician-diagnosed inhalant and food allergy were adapted from the International Study on Asthma and Allergy in Childhood (ISAAC) [13]. Current asthma (no; yes) was defined as ever diagnosis of asthma with wheezing or medication use in the past 12 months at 10 years of age. Parental reported questionnaires were used to define physician-diagnosed inhalant allergy (“Was your child ever diagnosed by a physician with an allergy to pollen (hay fever)/house dust mite/cat/dog?”) (no; yes) and food allergy (“Was your child ever diagnosed by a physician with an allergy to cashew nut/peanut?”) (no; yes) at age 10 years. Additionally, information on allergic rhinitis, a more detailed question on inhalant allergy, was obtained by a parental reported questionnaire (“Did your child had any sneezing, running nose or stuffed nose in the last 12 months, even though he or she did not have a cold or flu?” (no; yes). Information on allergic sensitization was collected by skin prick tests using the scanned area method [14, 15]. We examined the most prevalent food allergens for children at age 10 years at a population-based level, and therefore allergens for milk and egg were excluded [16, 17]. Inhalant allergens included house dust mite, 5-grass mixture, birch, cat, and dog. Food allergens included hazelnut, cashew nut, peanut and peach. Details on the collection of lung function, asthma and allergy measures are provided in the Additional file 1.

### Covariates

Information on parity, maternal education, and parental history of eczema, allergy or asthma was available from parental questionnaires obtained at enrolment. Child’s sex was obtained from midwives and hospital records, and ethnic origin based on the parents’ country of birth according to Statistics Netherlands [18]. Postnatal questionnaires provided information on breastfeeding at 2, 6 or 12 months after birth.

### Statistical analysis

Linear, logistic and multinomial regression models were used to examine the association of eczema phenotypes with lung function measures, risk of asthma, allergic sensitization or physician-diagnosed allergy, and combined allergic outcomes, respectively, using the packages ‘mice’ (version 3.3.0), ‘stats’ (version 3.5.2) and ‘nnet (version 7.3–12) in R version 3.5.2 [19–21]. The analyses were adjusted for potential confounders, selected from literature if they were related with both eczema phenotypes and the outcome and were not in the causal pathway. In order to examine inhalant allergies in detail, we also examined the correlation between physician-diagnosed inhalant allergy and allergic rhinitis, and the associations of eczema phenotypes with allergic rhinitis. To study the role of ethnicity in more detail, we performed a sensitivity analysis by stratifying for ethnicity (European or non-European). We only present the results based on imputed data, because the size and direction of effects were similar in complete-case-analysis. We did not adjust for multiple testing, because the respiratory and allergic measures were related to each other, and examined under the same hypothesis. More information on the statistical analyses is provided in the Additional file 1. All measures of association are presented as pooled z-score change or odds ratios (OR) with their corresponding 95% confidence intervals (95% CI).

## Results

### Subject characteristics

**Fig. 1:**
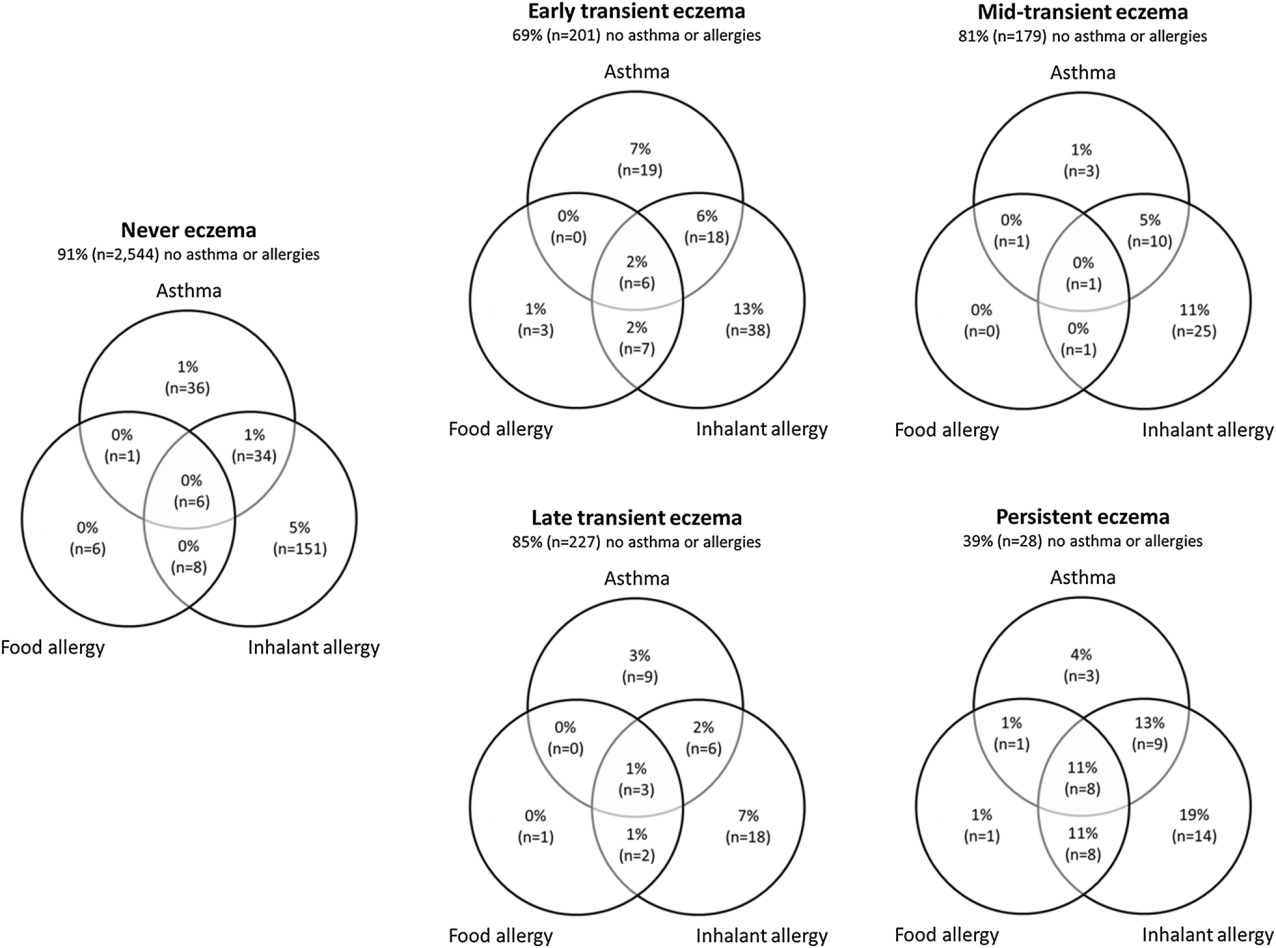
Prevalence of current asthma, physician diagnosed food and inhalant allergy in eczema phenotype. Values are percentages (absolute values) and based on observed data. n = number of participants with information on current asthma or physician diagnosed allergies, and at least 3 eczema measurements

Characteristics of children and their mothers are summarized in Table 1. For each eczema phenotype, the prevalence of current asthma, physician-diagnosed food allergy and inhalant allergy are presented in Fig. 1. Co-occurrence of these comorbidities was most prevalent in the persistent eczema group (range 1–19%). Main results of loss-to-follow-up analysis showed that children not included in the analyses more often had mothers of younger age, multiparity, lower education and no history of eczema, allergy or asthma, and more often had lower birth weight, a male sex and a non-European ethnicity mostly of Moroccan, Turkish and Cape Verdean ethnicity (Additional file 1: Table S1).

**Table 1:** Characteristics of children and their mothers

	Subjects n = 4277
Maternal characteristics	
Age at enrollment, years mean (SD)	31.7 (4.5)
Parity, nulliparous % (n)	59 (2526)
Maternal education, higher % (n)	59 (2510)
History of eczema, allergy and asthma, yes % (n)	61 (2597)
Child characteristics	
Sex, female % (n)	51 (2181)
Gestational age at birth, weeks median (2.5–97.5th percentile)^a^	40.1 (35.5–42.3)
Birth weight, grams mean (SD)^a^	3443.1 (566.9)
Ethnicity, non-European % (n)	24 (1006)
Breastfeeding, ever % (n)	93 (3961)
Eczema, ever % (n)^a^	23 (859)
Eczema phenotypes % (n)	
Never	76 (3229)
Early transient	9 (363)
Mid-transient	6 (259)
Late transient	8 (333)
Persistent	2 (93)
Current asthma, yes % (n)^b^	5 (203)
Inhalant sensitization, yes % (n)^b^	32 (985)
Food sensitization, yes % (n)^b^	7 (209)
Physician diagnosed inhalant allergy, yes % (n)^b^	12 (447)
Allergic rhinitis, yes % (n)^a^	20.6 (734)
Physician diagnosed food allergy, yes % (n)^b^	2 (79)
Lung function, Z-scores mean (SD)^a^	
FVC	0.18 (0.91)
FEV_1_	0.13 (0.96)
FEV_1_/FVC	− 0.12 (0.95)
FEF_75_	− 0.00 (0.91)

Values are percentages (absolute values), mean (SD) or median (2.5–97.5th percentile) after imputation. ^a^Data was missing and not imputed for gestational age at birth (0.2%), birth weight (0.1%), ever eczema (11.6%), allergic rhinitis (26.9%), and lung function (11.5%). ^b^Data on the following outcomes were not imputed for the individual analysis and were missing for: current asthma (9.7%), inhalant (26.9%) and food sensitization (27.1%), physician diagnosed inhalant (10.9%) and food allergy (12.7%). They were imputed for the combined outcome analysis and values are for current asthma (yes) 6% (n = 237), inhalant sensitization (yes) 33% (n = 1394), food sensitization (yes) 8% (n = 336), physician-diagnosed inhalant allergy (yes) 12% (n = 521) and physician-diagnosed food allergy (yes) 3% (n = 105)

### Eczema phenotypes, lung function and current asthma

Compared with never eczema, ever eczema was associated with a higher FVC and FEV_1_ (Z score change (95% CI): 0.08 (0.01, 0.16) to 0.08 (0.00, 0.16), respectively), but not with FEV_1_/FVC and FEF_75_. Ever eczema was associated with an increased risk of current asthma (OR (95% CI): 6.38 (4.61, 8.83) (Table 2). When examining eczema phenotypes, we observed that compared with the never eczema phenotype, only late transient eczema was associated with a higher FVC (Z score change (95% CI) 0.11 (0.00, 0.21)) (Table 2). All eczema phenotypes were associated with an increased risk of current asthma at the age of 10 years with the strongest effect estimates for early transient and persistent eczema (OR (95% CI) 4.82 (3.29, 7.08) and 11.53 (6.65, 20.01)). Similar size and direction of effect estimates were observed among children of European and non-European ethnicity (Additional file 1: Tables S2 and S3).

**Table 2:** Associations of eczema phenotypes with lung function and current asthma in children at age 10 years

	FVC Z-score (95% CI)	FEV_1_ Z-score (95% CI)	FEV_1_/FVC Z-score (95% CI)	FEF_75_ Z-score (95% CI)	Current asthma at 10 years odds ratio (95% CI)
Never eczema	Reference	Reference	Reference	Reference	Reference
Ever eczema	*0.08 (0.01, 0.16)*	*0.08 (0.00, 0.16)*	0.00 (− 0.07, 0.08)	0.02 (− 0.05, 0.09)	*6.38 (4.61, 8.83)*
Never	Reference	Reference	Reference	Reference	Reference
Early transient	0.04 (− 0.07, 0.14)	0.00 (− 0.10, 0.11)	− 0.02 (− 0.23, 0.19)	− 0.05 (− 0.15, 0.05)	*4.82 (3.29, 7.08)*
Mid-transient	− 0.07 (− 0.20, 0.05)	− 0.08 (− 0.21, 0.05)	− 0.04 (− 0.15, 0.07)	− 0.03 (− 0.15, 0.09)	*2.68 (1.58, 4.57)*
Late transient	0.11 (0.00, 0.21)	0.05 (− 0.06, 0.16)	− 0.11 (− 0.22, 0.00)	− 0.03 (− 0.13, 0.08)	*3.07 (1.94, 4.87)*
Persistent	0.04 (− 0.16, 0.24)	0.00 (− 0.21, 0.21)	− 0.02 (− 0.23, 0.19)	− 0.00 (− 0.20, 0.19)	*11.53 (6.65, 20.01)*

Values are Z-score mean differences for lung function measurements and odds ratios (95% confidence intervals) for current asthma from linear and logistic regression models for never/ever eczema. Values are average Z-score mean differences for lung function measurements and average odds ratios (95% confidence intervals) for current asthma from linear and logistic regression models, respectively, after multiple sampling based on 150 imputed datasets for eczema phenotypes. Lung function outcomes are force expiratory volume in 1 second (FEV_1_), forced vital capacity (FVC), force expiratory flow at 75% of the exhaled FVC (FEF_75_). Full models were adjusted for parental history of allergy, asthma or eczema, maternal education, parity, child’s sex, ethnicity and breastfeeding. Italic values indicate statistical significance at the α = 0.05 level

### Eczema phenotypes, allergic sensitization and physician-diagnosed allergies

Compared with never eczema, ever eczema was associated with increased risks of allergic sensitization and physician-diagnosed allergies for both inhalant and food allergens. The strongest association was observed for ever eczema with physician diagnosed food allergy (OR (95% CI) 11.89 (6.85, 20.61)) (Table 3). Of the eczema phenotypes, the early transient and persistent phenotypes were most strongly associated with increased risks of inhalant allergic sensitization (OR (95% CI) 2.62 (2.01, 3.42) and 4.53 (2.65, 7.51)), food allergic sensitization (OR (95% CI) 5.73 (3.94, 8.31) and 12.64 (7.20, 22.18)), physician-diagnosed inhalant allergy (OR (95% CI) 3.72 (2.78, 4.97) and 11.91 (7.52, 18.86)) and physician-diagnosed food allergy (OR (95% CI) 6.95 (3.76, 12.84) and 35.05 (18.33, 70.00)) (Table 3). Physician-diagnosed inhalant allergy and allergic rhinitis were correlated (Cramer’s V (Chi square *p* value) 0.50 (≤ 0.001)). The observed effect estimates of the associations of eczema phenotypes with allergic rhinitis were in the same direction, but less greater, versus those of eczema phenotypes with physician-diagnosed inhalant allergy (OR range (95% CI) 1.43 (1.02, 2.00) and 4.91 (3.14, 7.66) versus 1.92 (1.34, 2.74) and 11.91 (7.52, 18.86), respectively) (Additional file 1: Table S4). Similar size and direction of effect estimates were observed among children of European and non-European ethnicity (Additional file 1: Tables S2 and S3). Effect estimates were in the same direction and stronger if a child had both allergic sensitization and physician-diagnosed allergy (Additional file 1: Table S5).

**Table 3:** Associations of eczema phenotypes with allergic sensitization and physician-diagnosed allergies in children at age 10 years

	Inhalant sensitization odds ratio (95% CI)	Food sensitization odds ratio (95% CI)	Physician-diagnosed inhalant allergy odds ratio (95% CI)	Physician-diagnosed food allergy odds ratio (95% CI)
Never eczema	Reference	Reference	Reference	Reference
Ever eczema	*2.91(2.41, 3.52)*	*4.90 (3.60, 6.67)*	*4.54 (3.65, 5.63)*	*11.89 (6.85, 20.61)*
Never	Reference	Reference	Reference	Reference
Early transient	*2.62 (2.01, 3.42)*	*5.73 (3.94, 8.31)*	*3.72 (2.78, 4.97)*	*6.95 (3.76, 12.84)*
Mid-transient	*1.72 (1.25, 2.36)*	*2.13 (1.21, 3.76)*	*2.66 (1.86, 3.80)*	1.44 (0.43, 4.80)
Late transient	*1.77 (1.33, 2.35)*	*2.52 (1.56, 4.07)*	*1.92 (1.34, 2.74)*	*4.50 (2.19, 9.28)*
Persistent	*4.53 (2.65, 7.51)*	*12.64 (7.20, 22.18)*	*11.91 (7.52, 18.86)*	*35.05 (18.33, 70.00)*

Values are odds ratios (95% confidence intervals) from logistic regression models for never/ever eczema and average odds ratios (95% confidence intervals) from logistic regression models after multiple sampling based on 150 imputed datasets for eczema phenotypes. Full models were adjusted for parental history of allergy, asthma or eczema, maternal education, parity, child’s sex, ethnicity and breastfeeding. Italic values indicate statistical significance at the α = 0.05 level

### Eczema phenotypes, asthma and physician-diagnosed allergy combined

Compared with never eczema, ever eczema was associated with increased risks of both asthma only and physician-diagnosed allergy only (OR (95% CI) 5.83 (3.49, 9.74) and 4.03 (3.17, 5.11)), and most strongly with asthma and physician-diagnosed allergy combined (8.98 (5.89, 13.69)) (Table 4). Compared with never eczema phenotypes, early transient and persistent eczema were most strongly associated with asthma only (OR (95% CI) 5.36 (3.07, 9.36) and 5.23 (1.55, 17.63)), physician-diagnosed allergy only (3.68 (2.67, 5.08) and 10.02 (5.92, 16.96)), and asthma and physician-diagnosed allergy combined (7.11 (4.33, 11.67) and 29.03 (15.27, 55.22)). Effect estimates for eczema phenotypes were in the same direction and higher odds were observed when physician-diagnosed food and inhalant allergy were combined and when physician-diagnosed food and inhalant allergies were combined with asthma (Additional file 1: Table S6).

**Table 4:** Association of eczema phenotypes with combined asthma and physician-diagnosed allergy groups in children at age 10 years

	Asthma, but no allergy n = 97	Allergy, but no asthma n = 413	Asthma and allergy n = 140
Never eczema	Reference	Reference	Reference
Ever eczema	*5.83 (3.49, 9.74)*	*4.03 (3.17, 5.11)*	*8.98 (5.89, 13.69)*
Never	Reference	Reference	Reference
Early transient	*5.36 (3.07, 9.36)*	*3.68 (2.67, 5.08)*	*7.11 (4.33, 11.67)*
Mid-transient	1.37 (0.45, 4.19)	*2.21 (1.47, 3.32)*	*4.31 (2.33, 7.99)*
Late transient	*2.94 (1.47, 5.89)*	*1.76 (1.18, 2.64)*	*3.48 (1.88, 6.44)*
Persistent	*5.23 (1.55, 17.63)*	*10.02 (5.92, 16.96)*	*29.03 (15.27, 55.22)*

Values are odds ratios (95% confidence intervals) from logistic regression models for never/ever eczema and average odds ratios (95% confidence intervals) from multinomial regression models after multiple sampling based on 150 imputed datasets for eczema phenotypes. Reference group is no asthma and no allergy (n = 3627). n = number of participants with information on at least 3 eczema measurements. Missing data on asthma and physician-diagnosed allergy was imputed. Full models were adjusted for parental history of allergy, asthma or eczema, maternal education, parity, child’s sex, ethnicity and breastfeeding. Italic values indicate statistical significance at the α = 0.05 level

## Discussion

In this multi-ethnic population-based prospective cohort study, eczema phenotypes were differentially associated with the risk of allergic and respiratory conditions in school-aged children. The early transient and persistent eczema phenotypes were most consistently associated with asthma, allergic sensitization, and physician-diagnosed allergies, including allergic rhinitis. Results were similar for children of European and non-European ethnicity. Stronger effect estimates were observed for early transient and persistent eczema phenotypes with food allergy related measures and combined asthma and physician-diagnosed allergies. Compared with never eczema, ever eczema was associated with higher FVC and FEV_1_, but not with FEV_1_/FVC. Eczema phenotypes were not associated with any lung function measurement.

### Comparison with previous studies

When comparing results with previous studies, the difference in eczema phenotype definition and follow-up duration need to be taken into account. Previous cohort studies showed that children with early-onset and persistent eczema phenotypes have increased risks of asthma at ages 6 to 13 year [6, 7]. Results for mid- and late transient eczema phenotypes and the risk of asthma are inconsistent. Our observations in a multi-ethnic population are in line with previous findings and support that children with any eczema phenotype, but especially those with early onset and persistent eczema have increased risks of asthma at school-age [22, 23]. While eczema is strongly related to asthma and therefore hypothetically also with altered lung function, the relationship between eczema and lung function has not been studied. We observed that children with ever eczema had slightly higher FEV_1_ and FVC, but no changes in FEV_1_/FVC. These findings might be incidental, since there were no associations of eczema phenotypes with lung function measures. Other mechanisms might underlie the observed associations of ever eczema and eczema phenotypes with asthma, such as inhalant allergies and possible modulating effects of early allergic sensitization and allergic rhinitis [24]. Also all children included in our analysis had higher FEV_1_ and FVC z-scores, which might be explained by a relatively healthy study population or well-controlled asthma.

Previous studies showed that persistent eczema was associated with elevated total Immunoglobulin E levels at ages 7–8 years, and with an increased risk of sensitization to inhalant allergens, but not to food allergens at age 6 years [6, 7]. We showed that children with early transient and persistent eczema phenotypes had both allergic sensitization and physician-diagnosed allergies, with the strongest effect estimates for food allergy at age 10 years. These observed differences might be due to differences in number of children included for analysis, food allergy prevalence, eczema phenotypes definition and because our population has a longer follow-up which allowed the identification of more diverse phenotypes. A cohort study in children until age 6 years showed that children with early transient and persistent eczema had increased risks of food allergy and allergic rhinitis [7]. We observed similar results among children until age 10 years with allergic sensitization and physician-diagnosed food and inhalant allergies. Many children among the early transient and persistent eczema phenotype group had both asthma and multiple allergic conditions, and a large percentage of these (31–61%) had at least one diagnosis of asthma, food or inhalant allergy. Therefore, our results do not support the atopic march hypothesis in all children with eczema, but does show that in particular children with early transient and persistent eczema are likely to develop asthma and/or allergies later in childhood.

### Possible mechanisms

Early transient and especially persistent eczema consistently showed the strongest associations with asthma and allergic conditions. A common trait of both phenotypes is the early onset of eczema, suggesting that the period before the age of 2 years of age was important for the development of asthma and allergic conditions. Maturation rates of the skin, lungs and immune system from birth until 2 years are high and any change or disruption of these maturation processes might have long term consequences [25]. Proposed mechanisms include dysfunction of the epithelial barrier due to microbial and/or genetic factors and transcutaneous sensitization, leading to type 2 inflammation, and thereby predisposing to asthma and allergic conditions [25–27]. Our recent study showed an association of the four most common filaggrin mutations in Europeans with early and late transient eczema, but not with persistent eczema [10]. Unfortunately, we were not able to study filaggrin mutations as mediators for the association of eczema phenotypes with asthma and allergic conditions due to lack of power. Also sensitivity analysis in more detailed non-European ethnic subgroups was not possible due to small sample size. Therefore, future studies with larger sample sizes are needed to examine the potential mediating role of filaggrin mutations on the associations of eczema phenotypes with asthma and allergic conditions, and the role of different ethnicities.

### Strengths and limitations

The strengths of this study include the eczema phenotypes among a multi-ethnic population with detailed information on asthma, lung function, and multiple allergic conditions. By using multivariate regression models with multiple imputation and sampling we achieved more precise and unbiased effect estimates. However, some methodological considerations need to be taken into account. Children not included in the analyses partly had less favourable socio-economic factors and more often parents with no history or eczema, allergy or asthma. Selection bias due to lost to follow-up might have been present if the associations of eczema phenotypes with respiratory and allergic conditions were different in children that were not included in the analyses compared to the children that were included in the analyses. We aimed to minimize bias by imputation methods [20]. Despite validated questions, misclassification of eczema, asthma and physician diagnosed allergies remains possible due to self-response [13, 28]. We included the most relevant allergens for children of age 10 years at population level, and excluded allergens with low sensitization rates at this age, such as milk and egg [16, 17]. Residual confounding might be present since there might be factors not measured or not included in our analysis. For example, there was no information available to determine the severity of eczema. Furthermore, we were unable to perform our analyses in more detailed ethnic groups due to lack of power [29].

## Conclusion

Eczema phenotypes were differentially associated with risks of asthma and allergic conditions among school-aged children, and were similar in children from European and non-European ethnicity. The strongest and most consistent associations were found in children with early transient and persistent eczema. This suggests that children with early transient and persistent eczema might benefit from more intense follow-up for early identification and treatment of asthma and allergies.

## Supplementary information

**Additional file 1: Table S1.** Characteristics of children and their mothers of those included and not included in the analyses. **Table S2.** Associations of eczema phenotypes with asthma, allergic sensitization and physician-diagnosed allergies in children of European ethnicity at age 10 years. **Table S3.** Associations of eczema phenotypes with current asthma, allergic sensitization and physician-diagnosed allergies in children of non-European ethnicity at age 10 years. **Table S4.** Associations of eczema phenotypes with allergic rhinitis in children at age 10 years. **Table S5**. Association of eczema phenotypes with combined allergic sensitization and physician-diagnosed allergy groups in children at age 10 years. **Table S6.** Association of eczema phenotypes with combined asthma, physician-diagnosed inhalant and food allergy groups in children at age 10 years. **Figure S1.** Flow chart of participants included for analysis. **Figure S2.** Previously identified eczema phenotypes trajectories in 5297 children from latent class growth analysis.

## Data Availability

Data requests can be made to the secretariat of the Generation R Study.

## Abbreviations

ATS: American Thoracic Society
CI: Confidence interval
ERS: European Respiratory Society
FEF_75_: Forced expiratory flow after exhaling 75% of FVC
FEV_1_: Forced expiratory volume in 1 second
FVC: Forced vital capacity
ISAAC: International Study of Asthma and Allergies in Childhood
OR: Odds ratio
SPT: Skin prick test
