# Supplementary material for: Eczema phenotypes and risk of allergic and respiratory conditions in school age children
Source: Clin Transl Allergy. 2020 Feb 19;10:7. doi: 10.1186/s13601-020-0310-7 (PMC7029507; doi:10.1186/s13601-020-0310-7)

**Additional file materials**

**Eczema phenotypes and risk of allergic and respiratory conditions**

**in school age children**

Chen Hu^1,2,3^, Tamar Nijsten^2^, Evelien R. van Meel^1,3^, Nicole S. Erler^4^, Christophe Piketty^5^, Nicolette W. de Jong^6^, Suzanne G.M.A. Pasmans^2^, Johan C. de Jongste^3^, Liesbeth Duijts^3,7^

^1^The Generation R Study Group, Erasmus MC, University Medical Center Rotterdam, Rotterdam, The Netherlands. ^2^Department of Dermatology, Erasmus MC, University Medical Center Rotterdam, Rotterdam, The Netherlands. ^3^Department of Pediatrics, Division of Respiratory Medicine and Allergology, Erasmus MC, University Medical Center Rotterdam, Rotterdam, The Netherlands.^4^Department of Biostatistics, Erasmus MC, University Medical Center Rotterdam, Rotterdam, The Netherlands. ^5^CUTIS (Clinical Unit for Tests and Imaging of Skin), Evaluation Department, Nestlé Skin Health/ Galderma Research and Development, Sophia-Antipolis, France. ^6^Department of Internal Medicine, Division of Allergology & Clinical Immunology, Erasmus MC, University Medical Center Rotterdam, Rotterdam, The Netherlands. ^7^Department of Pediatrics, division of Neonatology, Erasmus MC, University Medical Center Rotterdam, Rotterdam, The Netherlands.

**Corresponding author**

Dr. Liesbeth Duijts, MD, PhD, Erasmus MC - Sophia, University Medical Center Rotterdam, Sp-3435; PO Box 2060, 3000 CB Rotterdam, The Netherlands. Tel: *31 10 7036263, Fax: *31 10 7036811, E-mail: l.duijts@erasmusmc.nl

**Methods**

Lung function, asthma and allergy

Spirometry was performed during the visit of the research center according to the American Thoracic Society (ATS) and European Respiratory Society (ETS) recommendations. Children with a >5% difference of FEV_1_ or FVC between curves, but at least one adequate curve according to ATS/ERS criteria were additionally included.

Allergic sensitization was defined by skin prick tests on the volar side of the left forearm with 2 positive (histamine) and 1 negative (saline) controls. The area of the wheal was measured 15 minutes after applying allergens to the skin using the Precise Automated Area Measurement of Skin Test (PAAMOST) software . The reaction was considered positive if the area of the wheal was ≥40% of the histamine response.

As allergic sensitization and physician-diagnosed allergy represent different clinical entities, we defined the following categories of allergy: 'no allergic sensitization and no physician-diagnosed allergy', 'any allergic sensitization, but no physician-diagnosed allergy', 'no allergic sensitization, but any physician-diagnosed allergy', and 'any allergic sensitization and any physician-diagnosed allergy'. Physician-diagnosed allergy and asthma were combined into four groups to examine the atopic burden in more detail: “no asthma and no allergy’, ‘asthma and no allergy’, ‘allergy and no asthma’ and ‘asthma and allergy’ and for a sensitivity analysis only physician-diagnosed allergy was further divided into inhalant and food allergy specific.

Statistical analysis

Pearson’s Chi-square, independent sample *t*- and Mann-Whitney U tests were used to compare characteristics of those included and not included in our study. We assumed that data were missing at random. Missing data in covariates were <20%. Combined asthma and allergy outcomes were imputed in the analysis to decrease bias and increase power. Using multiple imputation using chained equations, 150 datasets were created. For the eczema phenotypes, subjects were assigned to the latent classes based on their respective posterior probabilities . To take into account the uncertainty in class assignment, of each imputed dataset the class assignment was sampled using the subject specific posterior class probabilities determined by the latent class growth model . Analysis were performed on each of the 150 datasets separately and results were obtained by taking the mean and 2.5^th^ and 97.5^th^ quantiles of the parameter estimates.

References

1. van der Valk JP, Gerth van Wijk R, Hoorn E, Groenendijk L, Groenendijk IM, de Jong NW. Measurement and interpretation of skin prick test results. Clin Transl Allergy. 2015;6:8.

2. Hu C, Duijts L, Erler NS, Elbert NJ, Piketty C, Bourdes V, et al. Most associations of early-life environmental exposures and genetic risk factors poorly differentiate between eczema phenotypes: the Generation R Study. Br J Dermatol. 2019.

3. Schomaker M, Heumann C. Bootstrap inference when using multiple imputation. Stat Med. 2018.

**Table S1.** Characteristics of children and their mothers of those included and not included in the analyses

|  | **Included**  n=4,277 | **Not included**  n=3,116 | **p-value for difference** |
| --- | --- | --- | --- |
| **Maternal characteristics** |  | | |
| Age at enrollment, years mean (SD) | 31.7 (4.5) | 29.0 (5.5) | <0.001 |
| Parity % (n) |  |  |  |
| Nulliparous | 59 (2,468) | 51 (1,536) | <0.001 |
| Multiparous | 41 (1,696) | 49 (1,460) |  |
| Maternal education % (n) |  |  |  |
| Primary or secondary | 41 (1,677) | 71 (1,847) | <0.001 |
| Higher | 59 (2,446) | 29 (745) |  |
| History of eczema, allergy or asthma % (n) |  |  |  |
| No | 49 (2,010) | 55 (1,435) | <0.001 |
| Yes, at least one parent | 51 (2,092) | 45 (1,168) |  |
| **Child characteristics** |  |  |  |
| Sex % (n) |  |  |  |
| Male | 49 (2,096) | 52 (1,610) | 0.024 |
| Female | 51 (2,181) | 48 (1,505) |  |
| Gestational age at birth, weeks median (2.5-97.5th percentile) | 40.1 (35.5-42.3) | 40 (35.3-42.3) | <0.001 |
| Birth weight, grams mean (SD) | 3443.1 (566.9) | 3363.2 (577.9) | <0.001 |
| Ethnicity % (n) |  |  |  |
| European | 76 (3,252) | 51 (1,467) | <0.001 |
| Non-European | 24 (1,011) | 49 (1,416) |  |
| Breastfeeding % (n) |  |  |  |
| Never | 7 (303) | 11 (161) | <0.001 |
| Ever | 93 (3,858) | 90 (1,377) |  |
| Eczema phenotypes % (n) |  |  | 0.586 |
| Never | 76 (3,229) | 77 (470) |  |
| Early transient | 9 (363) | 8 (48) |  |
| Mid-transient | 6 (259) | 5 (29) |  |
| Late transient | 8 (333) | 8 (47) |  |
| Persistent | 2 (93) | 3 (17) |  |
| Eczema % (n) |  |  | 1.000 |
| Never | 77 (2,923) | 77 (714) |  |
| Ever | 23 (859) | 23 (210) |  |
| Current asthma % (n)* |  |  | 0.045 |
| No | 95 (3,658) | 93 (406) |  |
| Yes | 5 (203) | 7 (97) |  |
| Inhalant sensitization % (n)* |  |  | 0.088 |
| No | 69 (2,142) | 65 (681) |  |
| Yes | 32 (985) | 35 (356) |  |
| Food sensitization % (n)* |  |  | 0.177 |
| No | 93 (2,908) | 92 (951) |  |
| Yes | 7 (209) | 8 (86) |  |
|  |  |  |  |
| Physician diagnosed inhalant allergy % (n)* |  |  | 0.100 |
| No | 88(3,362) | 87 (804) |  |
| Yes | 12 (447) | 14 (125) |  |
| Allergic rhinitis % (n)* |  |  | <0.001 |
| No | 79.4 (2,837) | 73.2 (698) |  |
| Yes | 20.6 (734) | 26.8 (255) |  |
| Physician diagnosed food allergy % (n)* |  |  | 0.088 |
| No | 98 (3655) | 98 (886) |  |
| Yes | 2 (79) | 2 (19) |  |
| Lung function, Z-scores mean (SD)* |  |  |  |
| FVC | 0.18 (0.91) | 0.22 (1.01) | 0.018 |
| FEV_1_ | 0.13 (0.96) | 0.20 (1.04) | 0.125 |
| FEV_1_/FVC | -0.12(0.95) | -0.06 (1.01) | 0.037 |
| FEF_75_ | -0.00 (0.91) | 0.10 (0.98) | <0.001 |

Values are percentages (absolute values), mean (SD) or median (2.5-97.5th percentile) based on observed data.

**Table S2.** Associations of eczema phenotypes with asthma, allergic sensitization and physician-diagnosed allergies in children of European ethnicity at age 10 years

|  | **Current asthma**  **Odds Ratio (95%CI)**  n=3,021 | **Inhalant sensitization**  **Odds ratio (95% CI)** n=2,355 | **Food sensitization**  **Odds ratio (95% CI)** n=2,349 | **Physician-diagnosed inhalant allergy**  **Odds ratio (95% CI)** n=2,996 | **Physician-diagnosed food allergy**  **Odds ratio (95% CI)** n=2,941 |
| --- | --- | --- | --- | --- | --- |
|  |
| Never eczema | Reference | Reference | Reference | Reference | Reference |
| n= 2,319 | n= 2,318/2,319 | n= 1,708/2,319 | n= 1,707/2,319 | n= 2,292/2,319 | n= 2,260/2,319 |
| Ever eczema | **5.38 (3.69,7.85)** | **3.05 (2.49, 3.73)** | **4.84 (3.48,6.73)** | **4.86 (3.77,6.25)** | **10.06 (5.33, 18.99)** |
| n= 663 | n= 615/663 | n= 471/663 | n= 466/663 | n= 645/663 | n= 626/663 |
| Never | Reference | Reference | Reference | Reference | Reference |
| n=2,490 | n=2,320/2,490 | n=1,809/2,490 | n=1,807/2,490 | n=2,277/2,490 | n= 2,244/2,490 |
| Early transient | **5.12 (3.27, 8.03)** | **2.97 (2.22, 3.96)** | **6.00 (3.93, 9.15)** | **4.36 (3.14, 6.05)** | **6.47 (3.14,13.30)** |
| n=274 | n=247/274 | n=190/274 | n=186/274 | n=250/274 | n=240/274 |
| Mid-transient | **3.15 (1.75, 5.67)** | **1.82 (1.28, 2.61)** | **2.60 (1.40, 4.85)** | **3.18 (2.12, 4.75)** | 1.90 (0.56, 6.46) |
| n=204 | n=184/204 | n=140/204 | n=140/204 | n=189/204 | n=183/204 |
| Late transient | **3.16 (1.81, 5.49)** | **1.70 (1.22, 2.35)** | **2.63 (1.52, 4.54)** | **3.18 (1.29, 3.07)** | **3.94 (1.66, 9.36)** |
| n=244 | n=219/244 | n=175/244 | n=175/244 | n=222/244 | n=218/244 |
| Persistent | **11.71 (6.05,22.70)** | **5.31 (2.88, 9.79)** | **15.03 (7.98, 28.31)** | **15.83 (9.08, 27.59)** | **33.45 (15.39, 72.69)** |
| n=59 | n=51/59 | n=41/59 | n=41/59 | n=58/59 | n=56/59 |

Values are odds ratios (95% confidence intervals) from logistic regression models for never/ever eczema and average odds ratios (95% confidence intervals) from logistic regression models after multiple sampling based on 150 imputed datasets for eczema phenotypes. n = number of participants with information on current asthma, allergic sensitization or physician diagnosed allergies and at least 3 eczema measurements. Full models were adjusted for parental history of allergy, asthma or eczema, maternal education, parity, child’s sex and breastfeeding. Bold values indicate statistical significance at the α=0.05 level.

**Table S3. Associations of eczema phenotypes with current asthma, allergic sensitization and physician-diagnosed allergies in children of non-European ethnicity at age 10 years**

|  | **Current asthma**    **Odds Ratio (95%CI)** n=840 | **Inhalant sensitization**  **Odds ratio (95% CI)**  n=772 | **Food sensitization**  **Odds ratio (95% CI)**  n=768 | **Physician-diagnosed inhalant allergy**  **Odds ratio (95% CI)**  n=813 | **Physician-diagnosed**  **food allergy**  **Odds ratio (95% CI)**  n=793 |
| --- | --- | --- | --- | --- | --- |
|  |
| Never eczema | Reference | Reference | Reference | Reference | Reference |
| n=604 | n= 604/604 | n= 482/604 | n= 480/604 | n= 591/604 | n= 581/604 |
| Ever eczema | **9.44 (4.89,18.22)** | **1.98 (1.37,2.86)** | **3.43 (2.03, 5.79)** | **3.58 (2.37, 5.41)** | **13.21 (4.43,39.34)** |
| n=196 | n= 177/196 | n= 139/196 | n= 137/196 | n= 185/196 | n= 181/196 |
| Never | Reference | Reference | Reference | Reference | Reference |
| n=739 | n=628/739 | n=582/739 | n=579/739 | n=599/739 | n=588/739 |
| Early transient | **4.88 (2.43, 9.78)** | **2.08 (1.26, 3.45)** | **4.17 (2.22, 7.83)** | **2.56 (1.45, 4.51)** | **6.22 (2.12, 18.26)** |
| n=89 | n=67/89 | n=66/89 | n=66/89 | n=66/89 | n=65/89 |
| Mid-transient | 1.26 (0.36, 4.39) | 1.40 (0.75, 2.61) | 0.96 (0.25, 3.71) | 1.51 (0.70, 3.27) | 0.00 (0.00, >100) |
| n=55 | n=49/55 | n=42/55 | n=42/55 | n=50/55 | n=48/55 |
| Late transient | **2.79 (1.25, 6.23)** | **1.79 (1.07, 3.02)** | 2.14 (0.99, 4.66) | **1.88 (1.01, 3.49)** | **5.11 (1.55, 16.86)** |
| n=89 | n=71/89 | n=60/89 | n=60/89 | n=70/89 | n=65/89 |
| Persistent | **6.61 (2.69, 16.27)** | **4.54 (1.87, 11.00)** | **9.18 (3.77, 22.37)** | **6.25 (2.83, 13.76)** | **20.41 (6.69, 62.29)** |
| n=34 | n=25/34 | n=22/34 | n=21/34 | n=28/34 | n=27/34 |

Values are odds ratios (95% confidence intervals) from logistic regression models for never/ever eczema and average odds ratios (95% confidence intervals) from logistic regression models after multiple sampling based on 150 imputed datasets for eczema phenotypes. n = number of participants with information on current asthma, allergic sensitization or physician diagnosed allergies and at least 3 eczema measurements. Full models were adjusted for parental history of allergy, asthma or eczema, maternal education, parity, child’s sex and breastfeeding. Bold values indicate statistical significance at the α=0.05 level.

**Table S4.** Associations of eczema phenotypes with allergic rhinitis in children at age 10 years

|  | **Allergic rhinitis**  **Odds ratio (95% CI)** |
| --- | --- |
|  |
| Never eczema | Reference |
| Ever eczema | **2.73 (1.93, 3.86)** |
| Never | Reference |
| Early transient | **2.30 (1.77, 2.99)** |
| Mid-transient | **1.43 (1.02, 2.00)** |
| Late transient | **1.55 (1.16,2.07)** |
| Persistent | **4.91 (3.14, 7.66)** |

Values are odds ratios (95% confidence intervals) from logistic regression models for never/ever eczema and average odds ratios (95% confidence intervals) from logistic regression models after multiple sampling based on 150 imputed datasets for eczema phenotypes. Full models were adjusted for parental history of allergy, asthma or eczema, maternal education, parity, child’s sex and breastfeeding. Bold values indicate statistical significance at the α=0.05 level.

**Table S5**. Association of eczema phenotypes with combined allergic sensitization and physician-diagnosed allergy groups in children at age 10 years.

|  | **Any allergic sensitization, but no allergy**  n= 1,005 | **No allergenic sensitization, but any allergy**  n= 101 | **Any allergic sensitization and any allergy**  n= 452 |
| --- | --- | --- | --- |
| Never eczema | Reference | Reference | Reference |
| n=2,923 | n=637/2,923 | n=33/2,923 | n=178/2,923 |
| Ever eczema | **2.15 (1.74, 2.64)** | **4.02 (2.17, 7.43)** | **6.28 (4.94, 7.99)** |
| n=859 | n=251/859 | n=24/859 | n=210/859 |
| Never | Reference | Reference | Reference |
| n=3,229 | n=714/3,229 | n=63/3,229 | n=223/3,229 |
| Early transient | **2.39 (1.77, 3.24)** | **3.55 (1.62, 7.78)** | **5.95 (4.29, 8.23)** |
| n=363 | n=114/363 | n=15/363 | n=93/363 |
| Mid-transient | 1.44 (0.99,2.08) | 2.40 (0.99, 5.79) | **2.97 (1.98, 4.46)** |
| n=259 | n=65/259 | n=9/259 | n=42/259 |
| Late transient | **1.63 (1.18, 2.25)** | 1.80 (0.76, 4.27) | **2.47 (1.68, 3.64)** |
| n=333 | n=96/333 | n=10/333 | n=45/333 |
| Persistent | **2.24 (1.11, 4.51)** | **4.93 (1.24, 19.56)** | **19.93 (11.42, 34.76)** |
| n= 93 | n=17/93 | n=4/93 | n=50/93 |

Values are odds ratios (95% confidence intervals) from logistic regression models for never/ever eczema and average odds ratios (95% confidence intervals) from logistic regression models multiple sampling on 150 imputed datasets for eczema phenotypes. Reference group are children without any allergic sensitization or physician-diagnosed allergy (n =2,719). n = number of participants with information on at least 3 eczema measurements. Missing data on allergic sensitization and physician-diagnosed allergy was imputed. Full models were adjusted for parental history of allergy, asthma or eczema, maternal education, parity, child’s sex, ethnicity and breastfeeding. Bold values indicate statistical significance at the α=0.05 level.

**Table S6.** Association of eczema phenotypes with combined asthma, physician-diagnosed inhalant and food allergy groups in children at age 10 years.

|  | **Inhalant allergy, but no asthma and no food allergy**  n=345 | **Food allergy, but no asthma and no inhalant allergy**  n=25 | **Inhalant and food allergy, but no asthma**  n=43 | **Asthma, but no inhalant and food allergy**  n=97 | **Asthma and inhalant allergy, but no food allergy**  n=103 | **Asthma and food allergy, but no inhalant allergy**  n=7 | **Asthma, inhalant and food allergy**  n=31 |
| --- | --- | --- | --- | --- | --- | --- | --- |
| Never  n=3,229 | **Reference** | **Reference** | **Reference** | **Reference** | **Reference** | **Reference** | **Reference** |
| Early transient  n=363 | **3.22 (2.27, 4.56)** | **5.72 (1.67,19.60)** | **9.03 (3.61, 22.57)** | **5.36 (1.55, 9.35)** | **6.07 (3.44, 10.71)** | 7.14 (0, >100) | **11.84 (3.82, 36.70)** |
| Mid-transient  n=259 | **2.37 (1.56, 3.59)** | 0 (0,>100) | 1.42 (0.19, 10.71) | 1.37 (0.45, 4.19) | **4.35 (2.20, 8.61)** | 10.15(0.68, >100) | 2.82 (0.36, 22.29) |
| Late transient  n=333 | 1.56 (0.92, 2.31) | 2.11 (0.34, 13.13) | **6.08 (2.22, 16.61)** | **2.94 (1.47, 5.90)** | **2.82 (1.34, 5.95)** | 0.00 (0, >100) | **7.24 (1.92, 27.24)** |
| Persistent  n=93 | **6.69 (3.63,12.33)** | **8.19 (1.04,64.52)** | **67.07(26.13,>100)** | **5.23 (1.55,17.64)** | **17.14 (7.68, 38.24)** | **67.57(4.60, >100)** | **97.86(31.97, >100)** |

Values are odds ratios (95% confidence intervals) from logistic regression models for never/ever eczema and average odds ratios (95% confidence intervals) from multinomial regression models after multiple sampling based on 150 imputed datasets for eczema phenotypes. Reference group is no asthma and no physician-diagnosed food and inhalant allergy (n=3,627). n = number of participants with information on at least 3 eczema measurements. Missing data on asthma and physician-diagnosed allergy was imputed. Full models were adjusted for parental history of allergy, asthma or eczema, maternal education, parity, child’s sex, ethnicity and breastfeeding. Bold values indicate statistical significance at the α=0.05 level.

**Figure S1.** Flow chart of participants included for analysis

**n = 7,393**

Prenatally included children with participation in postnatal phase

**n = 2,096**
Missing information on >3 eczema measures from birth until age 10 years

**n = 5,297**

Children with information on ≥3 eczema measures from birth until age 10 years available

**n = 1,020**
Missing information on all measures of allergic and respiratory conditions at age 10 years

**n = 4,277**

Children with information on any measure of allergic or respiratory conditions at age 10 years available

FVC n=3,790

FEV1 n=3,786

FEV1/FVC n=3,786

FEF75 n=3,789

Current asthma n=3,861

Inhalant sensitization n=3,127

Food sensitization n=3,117

Physician-diagnosed

inhalant allergy n=3,809

Physician-diagnosed

food allergy n=3,734

**Figure S2.** Previously identified eczema phenotypes trajectories in 5,297 children from latent class growth analysis


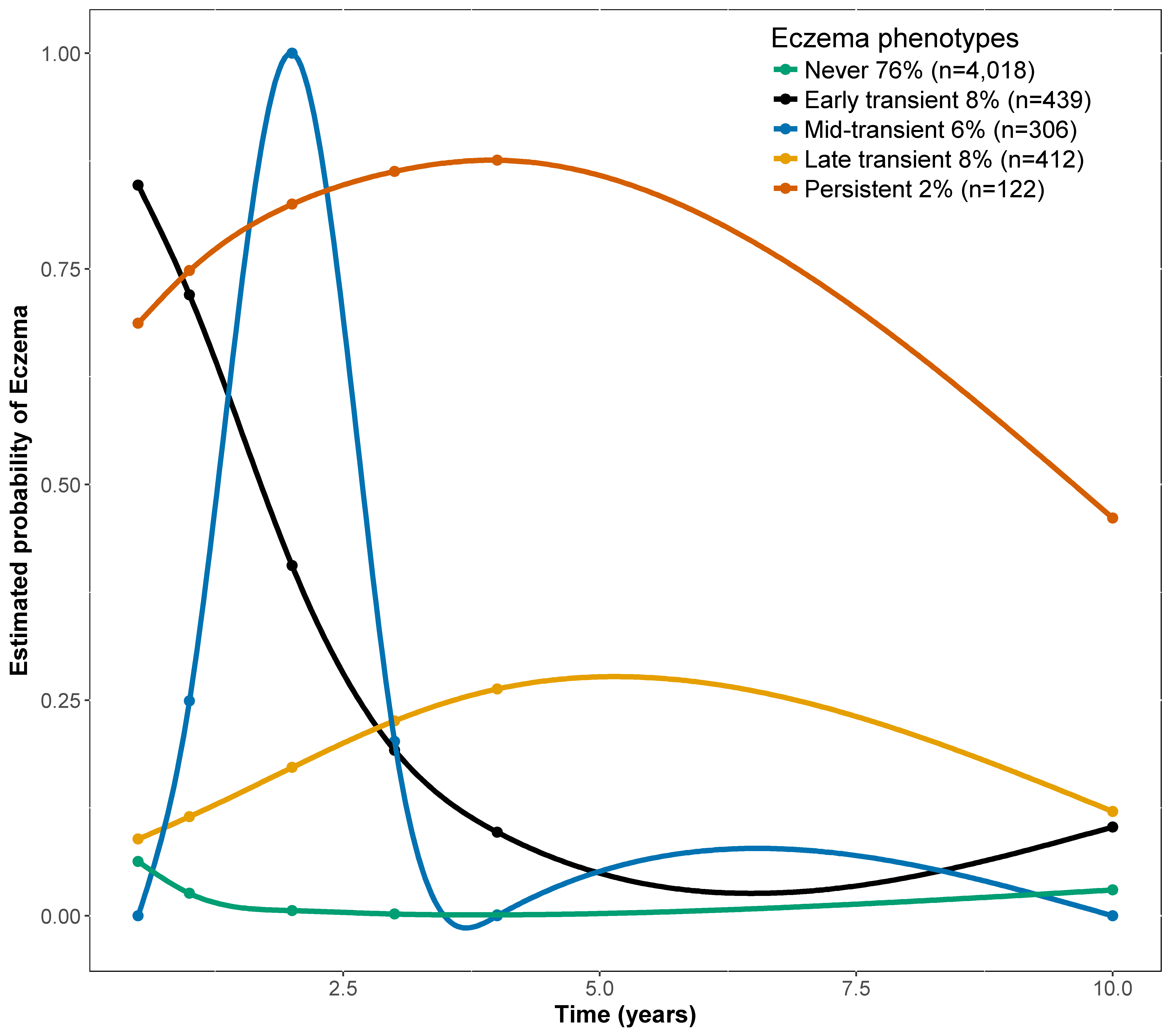

Supplement: Supplementary file 1 — Additional file 1: Table S1. Characteristics of children and their mothers of those included and not included in the analyses. Table S2. Associations of eczema phenotypes with asthma, allergic sensitization and physician-diagnosed allergies in children of European ethnicity at age 10 years. Table S3. Associations of eczema phenotypes with current asthma, allergic sensitization and physician-diagnosed allergies in children of non-European ethnicity at age 10 years. Table S4. Associations of eczema phenotypes with allergic rhinitis in children at age 10 years. Table S5. Association of eczema phenotypes with combined allergic sensitization and physician-diagnosed allergy groups in children at age 10 years. Table S6. Association of eczema phenotypes with combined asthma, physician-diagnosed inhalant and food allergy groups in children at age 10 years. Figure S1. Flow chart of participants included for analysis. Figure S2. Previously identified eczema phenotypes trajectories in 5297 children from latent class growth analysis. [file 13601_2020_310_MOESM1_ESM.doc]
